# Laparoscopic simultaneous resection of bilateral giant primary mature retroperitoneal teratoma of the adrenal region: A case report

**DOI:** 10.1097/MD.0000000000017836

**Published:** 2019-11-01

**Authors:** Jianqing Wang, Jibing Zhang, Chuan Xiao, Caibin Fan

**Affiliations:** a Department of Urology; b Department of Radiology; c Department of Pathology, The Affiliated Suzhou Hospital of Nanjing Medical University, PR China.

**Keywords:** laparoscopic simultaneous resection, teratoma

## Abstract

**Rationale:**

Giant mature retroperitoneal teratoma of the adrenal region is quite rare in adults. In most cases, open adrenalectomy is required to ensure complete resection. We describe a case of bilateral giant primary mature cystic teratoma in the region of both adrenal glands in a 22-year-old female patient.

**Patient concerns:**

A 22-year-old female patient was admitted to our hospital with no complain after detecting to have 2 giant well circumscribed masses in a routine investigation.

**Diagnoses:**

She was diagnosed with bilateral giant primary mature retroperitoneal teratoma of the adrenal region.

**Interventions:**

The patient underwent en bloc excision of the mass through laparoscopic simultaneous resection.

**Outcomes:**

We carefully separated and retained most of the adrenal tissue on both sides during surgery. Pathology reported mature teratomas. Eleven days after operation, the patient made uneventful recovery and left the hospital without any complication.

**Lessons:**

Preoperative imaging and histologic analysis confirmed mature retroperitoneal teratomas. It is feasible to treat such giant benign tumors by laparoscopic simultaneous resection.

## 1 Introduction

Teratomas are neoplasms of the embryonic tissues that typically arise in the gonads (testes and ovaries) and sacrococcygeal regions of adults and children. Mature teratomas are made up of well-differentiated parenchymal tissues composed of somatic cell types which are derived from 2 or more germlayers (ectoderm, mesoderm, or endoderm).^[1,2]^ Among them, retroperitoneal teratomas, including teratomas of adrenal region, account for only 4% of all primary teratomas, and are actually quite rare in adults.^[3]^ Until now, only a very few case reports have been documented in literature. In most cases, patients are asymptomatic, have no specific complaints or are identified on routine physical examination incidentally.^[4]^ Open or laparoscopic resection of the lesions is the mainstay of treatment, and can give us an opportunity to determine the definitive diagnosis by histopathological examination. In terms of prognosis, patients with complete lesion resection often have an excellent prognosis with an overall 5-year survival rate of nearly 100%.^[5]^ Bilateral giant mature teratoma of the adrenal region and the treatment strategy has not been documented. Here we report a case of bilateral giant primary mature cystic teratoma in the region of both adrenal glands in a young adult female patient treated by laparoscopic simultaneous resection for the first time.

## 2 Case report

A 22-year-old previously healthy female patient was found to have 2 giant well-circumscribed masses with rim shadowing in the region of both adrenal regions by abdominal ultrasound in a routine investigation. She was asymptomatic and then referred to our hospital for further examination and treatment. At physical examination, her arterial blood pressure was 128/77 mm Hg. Abdominal examination disclosed 2 global distention with dullness on the both sides of her upper abdomen. No other physical findings were observed. The baseline workups for this patient, including complete blood count, renal, and liver function tests, were within normal limits. Functional evaluations for adrenal hormones, including 24-hour urine cortisol, fractionated metanephrines, and plasma aldosterone to renin ratio, were also within normal limits.

**Figure 1:**
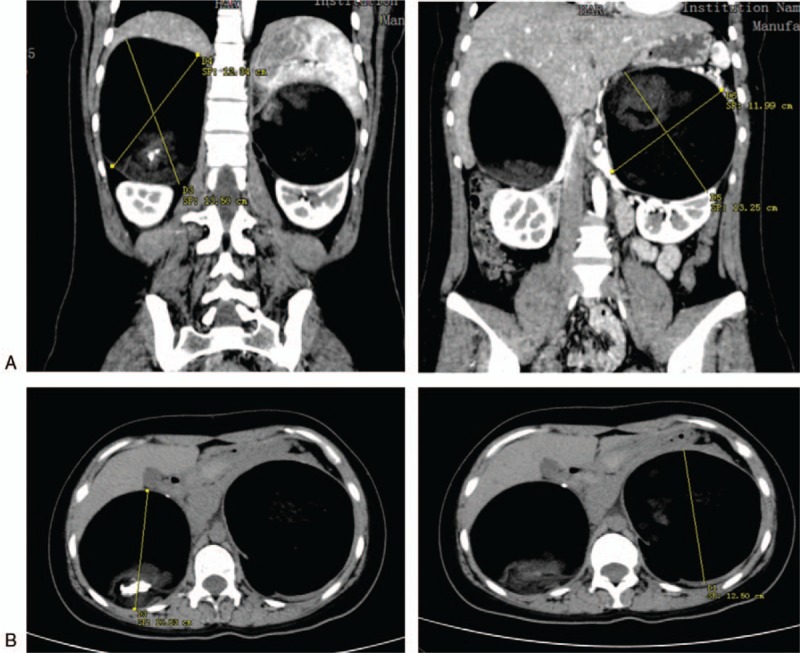
CT images of bilateral cystic teratoma: coronal view (A) and cross-sectional view (B).

**Figure 2:**
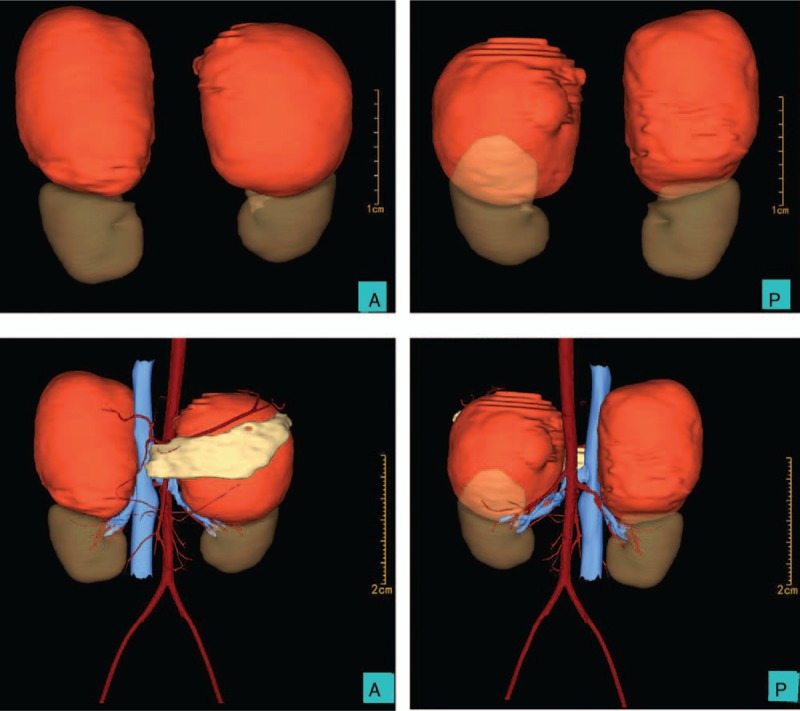
Three-dimensional digital reconstruction of kidneys, blood vessels, and masses based on CT images. Total tumor volume was 1698.6 ml, while the total volume of 2 kindeys was 347.6 ml.

During the course of investigations, an abdominal and pelvic computed tomography (CT) scan showed 10.4 × 10.1 × 13.2 cm and 12.3 × 10.5 × 12.5 cm solid and cystic masses in the left and right adrenal region containing bone and multiple soft tissue densities, respectively (Fig. 1). In order to better define the condition of the tumor and its relationship with the kidney, we did a three-dimensional digital reconstruction of the kidneys, blood vessels, and masses based on CT images. Total tumor volume was 1698.6 ml, while the total volume of 2 kidneys was 347.6 ml. Both of the 2 kidneys were squeezed and deformed and the pancreas was densely adherent to left mass (Fig. 2). There was no evidence of distant metastasis. In further assessment, abdominal magnetic resonance imaging (MRI) revealed a 10 × 10.8 × 12.9 cm mass and a 10.3 × 12.3 × 12.1 cm mass with heterogeneous signal intensity, heterogeneous enhancement, and predominantly fatty composition in the left and right adrenal region, respectively, suggestive of 2 adrenal teratomas. Considering that the masses are more likely to be benign tumors, that the patient is a young unmarried woman, and that we must retain normal adrenal tissue as much as possible to maintain the function, we decided to carry out laparoscopic simultaneous resection.

**Figure 3:**
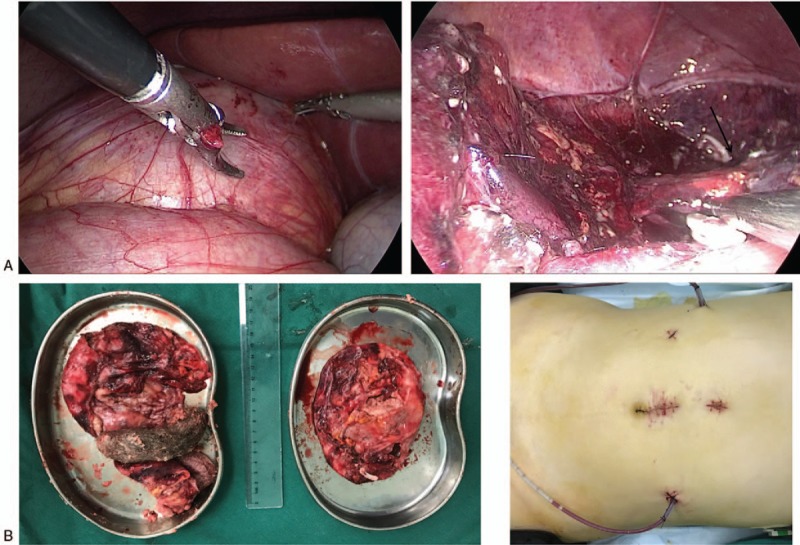
We reserved the adrenal gland (arrow) densely adherent to the giant teratoma at laparoscopic resection (A). We showed the bilateral cystic teratoma after excision (B). We used 5 operating holes with a 4 cm-incision to perform the operation (C).

The patient took a supine position and the waist was blocked up. A 4 cm incision was made upon the umbilicus and a 10-mm trocar was placed. A 12-mm Trocar was inserted 3 cm under the xiphoid process, and 2 5-mm Trocars were placed 3 cm below ribs on the right anterior axillary line and 1 cm below ribs on midaxillary line, respectively. We first dealt with the lesion on the right side. Although the mass was located in the region of right adrenal gland, we found that the adrenal gland was on the external surface of the mass and distinct from the mass. We separated the tumor carefully from the adrenal gland and retained most of the normal adrenal tissue (Fig. 3 A). Because of its large size, the tumor collapsed during careful dissection. We drained the mixed orange-light brown, fatty, and mucoid mass with black strands of hair and completely removed the tumor. On the left side, we added a 12-mm Trocar on the 3 cm below ribs on the left anterior axillary line. The tumor was densely adherent to the pancreas and aorta, which made the dissection more tedious and difficult. After careful dissection, we found the mass was densely adherent to, but still distinct from the left adrenal gland. The tumor also collapsed, and we drained the yellow-gray sebaceous substance and hair and completely removed the tumor. The left adrenal gland was retained entirely. Finally, the tumors were removed meanwhile and sent for histopathological evaluation (Fig. 3 B). During the whole operation, we only used 5 operating holes with a 4 cm-incision (Fig. 3 C). The operation time was about 480 minutes. The amount of bleeding was 1000 ml while the blood transfusion was 700 ml. The postoperative course was uneventful and the patient was discharged on postoperative day 11. The patient was free of recurrence after 10 months of follow-up.

**Figure 4:**
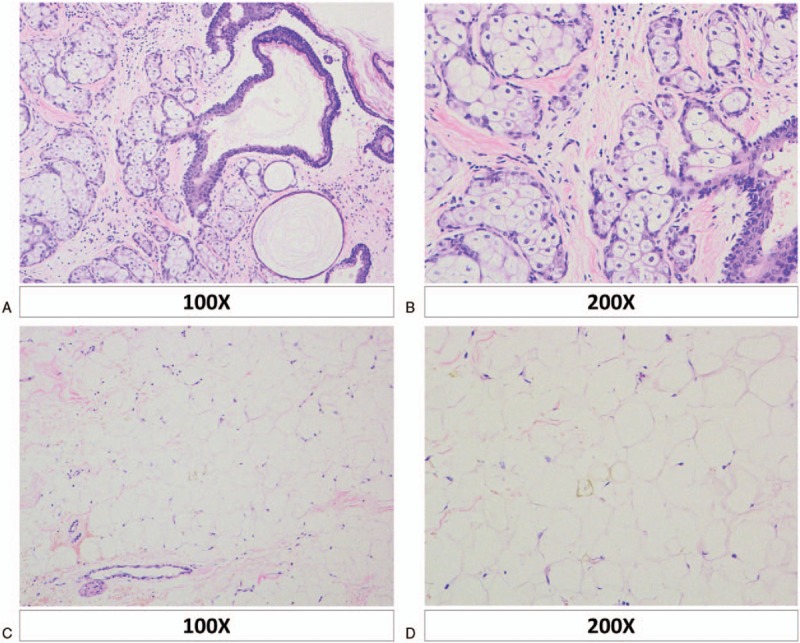
Photomicrograph of the tumor shows mature glandular epithelium, mucosal epithelium (A, B), and adipose (C, D) tissue in the wall (hematoxylin and eosin).

The final pathological evaluation of the retroperitoneal masses confirmed the diagnosis of mature teratomas, with abundant fat deposits and different tissues such as adipose tissue, skin, and sebaceous appendages identified in the cystic wall (Fig. 4). These findings confirmed the lesions to be benign mature cystic teratomas. Because these tumors usually represent metastasis from other primary sites, additional imaging with CT of the chest and abdomen, and ovarian ultrasonography was performed. No other primary tumor was identified. Therefore, we diagnosed the masses as primary retroperitoneal teratomas.

## 3 Discussion

Teratomas are rare neoplastic tumors derived from totipotential cells, composed of tissues from at least 2 embryological layers. In females of reproductive age (20–40 years), most of teratomas arise from the ovary, of which the incidence is 1.2 to 14.2 cases per 100,000 people per year.^[6]^ Cases of primary teratomas in the retroperitoneum usually occur in pediatric populations, but are rare in adults.^[7]^ This case is the first case of bilateral giant primary mature retroperitoneal teratoma reported in the literature so far. In previous artticles, retroperitoneal teratoma ≥10 cm in diameter called bulky tumor. The diameter of giant retroperitoneal tumor is between 57 and 200 mm, and the average is 100 mm.^[9]^ In sacrococcygeal teratoma, tumor >10 cm is defined as giant sacrococcygeal teratoma.^[10]^ In the present report, our patient has a 10 × 10.8 × 12.9 cm mass and a 10.3 × 12.3 × 12.1 cm mass. Both teratomas were quite large and could be defined as giant teratomas as indicated in previous articles and studies. Giant primary retroperitoneal teratomas are always diagnosed as myelolipomas at the first sight on the basis of the radiologic imaging. Previous investigations suggested that retroperitoneal teratomas, including teratomas in adrenal region, have very little malignant potential, and can be removed laparoscopically if they are not too large.^[8]^

The clinical presentations of teratomas are variable. The most common clinical presentations include nonspecific pain in the abdomen or back, obstructive gastrointestinal, and genitourinary symptoms, as well as lower limb/genital swelling resulting from lymphatic obstruction.^[9]^ Some patients can present with complications such as infections (abscess formation),^[10]^ traumatic rupture leading to acute peritonitis,^[11]^ or malignant transformations rarely.^[12]^ Midline teratoma masses with restricted mobility can be easily detected on physical examination. Besides, however, very a few patients are asymptomatic with no complain.^[13]^ In this study, our patient was asymptomatic, and was found to have masses by abdominal ultrasound in a routine investigation. Her teratomas was located in both adrenal regions and shares very similar radiological and histopathological features with the cases reported in other previous literatures.^[3,9,14,15]^

The preoperative diagnosis of a teratoma of adrenal region is difficult, because of the varied imaging characteristics based on the different composition of the mass. Radiographic investigations, including US, CT, and MRI, play valuable roles in diagnosing teratomas.^[13]^ Compared with other adrenal tumors (such as adrenal myelolipoma), the most characteristic imaging feature of mature teratomas is a more heterogeneous mass containing fluid, fat, and/or sebum in the form of a fat–fluid level and calcification, just as seen in our patient.^[16,17]^ Specifically, X-ray (plain radiographs) can identify calcified elements in about 62% of cases^[18–20]^ whereas ultrasound (US) can greatly distinguish between cystic and solid elements. The most valuable examinations might be CT and MRI. On CT or MRI, teratomas are frequently cystic and contain calcifications, including teeth. CT scans can easily differentiate between adipose tissue and bone (calcified) masses. By comparison, MRI scans show better resolution of soft tissues (adipose tissue), feasible identification of benign and malignant neoplastic features, and most importantly superior tumor staging assessment.^[21]^ However, it still remains challenging to distinguish teratoma from the more common fatty myelolipoma.^[22]^ Another aspect is the rarity of this disease. In this case, CT and MRI examinations of our patient both recommended diagnosing the masses as retroperitoneal teratomas rather than myelolipomas. In addition to their diagnostic role, such imaging studies provide better preoperative planning and increased likelihood of complete removal of the tumour with less iatrogenic damage. They can display the precise location, morphology, and adjacent structures of the teratoma, which is of utmost importance.^[23]^ Histologic analysis confirmed the diagnosis of teratomas, which showed the possibility that bilateral giant teratoma, other than myelolipoma, arises in adrenal regions in adults. This can help other similar cases in preoperative diagnosis, better preoperative planning, and patient counseling in the future.

As to the treatment, due to their large size, an open approach is often the chief approach to resect these giant masses when the teratomas are larger than 6 cm.^[24]^ One defect of laparotomy is the inability to find and preserve the adrenal tissue, which is always displaced and is densely adhered to the giant teratoma. This is especially vital in young adult patients with bilateral lesions. The lack of adrenal corticosteroid is fatal, and lifelong adrenal corticosteroid replacement therapy impairs patients’ life quality seriously. For benign tumors, laparoscopic resection can help surgeons overcome the problems above by offering a better operational view for careful dissection. As long as the tumor has no local invasion on preoperative imaging or is found intraoperative, laparoscopic resection can be performed safely and can replace open surgery in most cases.^[4]^ In addition, the advantages of laparoscopic resection include beautiful incision, small surgical damage and shorten recovery time after surgery. In this case, considering that the masses are more likely to be benign tumors, to retain normal adrenal tissue as much as possible to maintain the function, we performed laparoscopic simultaneous resection and provided a reference for the feasibility of this approach. Careful preoperative evaluation is the key step to help us determine the first operation side. In our opinion, starting from the relatively simple side can help preserve the adrenal tissue. After this, surgeons can handle the difficult side completely regardless of the possibility of adrenalectomy in this step. Of course, preoperative evaluation is the most important point for choosing surgical approach. Laparoscopic surgery is only suitable for benign tumors, which highlights the important role of imaging assessment and surgeon experience.

In terms of prognosis, mature teratomas are likely to be benign and carry an excellent prognosis with overall 5-year survival nearly 100% after treatment.^[5]^ However, regardless of the benign histological nature of mature teratomas and no findings elsewhere, close follow-up is still recommended because the incidence of malignant transformation is about 3% to 6%.^[3]^ In the present case, the patient recovered with no sign of recurrence after 10 months of follow-up.

From this case, we showed the feasibility of laparoscopic resection to deal with such bilateral giant mature retroperitoneal teratomas, which retains adrenal tissue more effectively. Starting from the relatively simple side is recommended. The limitations of this study include the low incidence and relatively lower accuracy in laparoscopic resection. Maybe more cases in future will help surgeons to deal with such giant teratomas more appropriately.

In conclusion, we report a bilateral giant primary mature retroperitoneal teratoma of the adrenal region for the first time. Preoperative imaging and histologic analysis confirmed mature retroperitoneal teratomas. We performed laparoscopic simultaneous resection to carry out en bloc excision and retained most of the adrenal tissue successfully, which suggests the feasibility of this approach. Further characterization of the cytogenetic changes underpinning tumorigenesis in such teratoma may provide additional insights into its prognosis.

## Author contributions

**Conceptualization:** Caibin Fan.

**Investigation:** Jianqing Wang, Caibin Fan.

**Methodology:** Jianqing Wang, Jibing Zhang, Chuan Xiao, Caibin Fan.

**Supervision:** Caibin Fan.

**Writing – original draft:** Jianqing Wang.

**Writing – review & editing:** Caibin Fan.

Jianqing Wang orcid: 0000-0002-0709-9694.
